# Supplementary material for: Resolving the mechanisms of hygroscopic growth and cloud condensation nuclei activity for organic particulate matter
Source: Nat Commun. 2018 Oct 4;9:4076. doi: 10.1038/s41467-018-06622-2 (PMC6172236; doi:10.1038/s41467-018-06622-2)
Supplement: Supplementary file 1 — Supplementary Information [file 41467_2018_6622_MOESM1_ESM.pdf]

Supplementary Information

Resolving the Mechanisms of Hygroscopic Growth and Cloud Condensation Nuclei Activity for Organic Particulate Matter

Liu et al.

Supplementary Methods

1. Thermodynamic Modeling

The observed thermodynamic behaviors of biogenically-derived SOM were simulated using a coupled Flory-Huggins-Köhler model framework. The Flory-Huggins solution theory was originally developed for describing the interaction between long-chain polymers with solutions[1, 2]. The Flory-Huggins model uses volume fractions instead of molar fractions, because the solute and solvent molecules can be very different in size. It is formulated as a free energy function, from which chemical potential of each compound can be derived. The free energy function includes contributions from both the entropic term of ideal mixing, and the enthalpic term of non-ideal interactions. The enthalpic term can be described by the interaction parameter χ. The original Flory-Huggins model with a constant χ does not account for the concentration-dependent interaction of hydrogen bonding for organics in water. This effect can be taken into account using a concentration-dependent χ[3].

Within the atmospheric sciences, Petters et al.[4] has coupled the Flory-Huggins theory to the Köhler theory to model the hygroscopic growth and cloud forming behaviors of atmospherically relevant oligomers and polymers. In the present work, this Flory-Huggins-Köhler framework has been further developed for modeling the thermodynamics of SOM with water. In brief, the original binary model was extended to a ternary form for modeling complex mixtures. Possible LLPS state and the composition for each liquid phase were calculated by minimizing the free energy. Surface tension of the gas-particle interface was calculated based on the predicted composition of particle surface. A minimum monolayer thickness of the outer shell phase was considered, similar to the treatment of organic film models in recent literature by

Ruehl et al.[5] and Ovadnevaite et al.[6], and the effect of partial surface coverage for surface tension was accounted.

1.1 Flory-Huggins Model for Water Activity

A detailed derivation for the ternary Flory-Huggins equations has been provided in ref.[7], and only a synopsis is provided herein. The general form of the free energy density function *f* for an aqueous solution can be expressed as:

$$\frac{fv_0}{RT} = \sum_i \frac{\phi_i}{N_i} \ln \phi_i + \sum_{i \neq j} \chi_{ij} \phi_i \phi_j \tag{S1}$$

where *R* is the gas constant, *T* is the absolute temperature, v_0 is the molar volume of water, v_i is the molar volume of compound *i*, ϕ_i is the volume fraction of compound *i*, $N_i = v_i / v_0$ is the ratio of molar volumes, and χ_{ij} is the Flory-Huggins interaction parameter between compounds *i* and *j*. The first and second terms on the right-hand side represent the entropic and enthalpic contributions, respectively.

The chemical potential of compound *i*, μ_i, can be derived using the equation below:

$$\hat{\mu}_i = f + (1 - \phi_i) \frac{\partial f}{\partial \phi_i} - \phi_j \frac{\partial f}{\partial \phi_j} \tag{S2}$$

where $\hat{\mu}_i = \mu_i / v_i$. For a ternary mixture, the chemical potential for water (compound 0) can be written as[7]:

$$\ln a_w = \frac{\hat{\mu}_0 v_0}{RT} = \ln(1 - \phi_1 - \phi_2) + \left(1 - \frac{1}{N_1}\right)\phi_1 + \left(1 - \frac{1}{N_2}\right)\phi_2 + (\chi_{01}\phi_1 + \chi_{02}\phi_2)(\phi_1 + \phi_2) - \chi_{12}\phi_1\phi_2 \tag{S3}$$

Subscription 0, 1, and 2 represent water, hydrophobic organic component, and hydrophilic organic component, respectively. We further assume that the hydrophobic and hydrophilic organics have the same molar volume, i.e., $N_1 = N_2 = N$, Eq. S3 can be simplified as:

$$\begin{aligned}\ln a_w &= \frac{\hat{\mu}_0 v_0}{RT} \\ &= \ln\left(1-\phi_1-\phi_2\right)+\left(1-\frac{1}{N}\right)\left(\phi_1+\phi_2\right) \\ &\quad +\left(\chi_{01}\phi_1+\chi_{02}\phi_2\right)\left(\phi_1+\phi_2\right)-\chi_{12}\phi_1\phi_2\end{aligned} \tag{S4}$$

For the binary case ($\phi = \phi_1$, and $\phi_2 = 0$), Eq. S3 can be reduced to:

$$\ln a_w = \frac{\hat{\mu}_0 v}{RT} = \ln\left(1-\phi\right)+\left(1-\frac{1}{N}\right)\phi+\chi\phi^2 \tag{S5}$$

which is consistent with the binary Flory-Huggins equation in literature [4]. ϕ-dependent interaction parameter χ between water and SOM can be obtained from the sorption isotherm measured by the QCM using Eq. S5. The measured values of χ, as illustrated in Supplementary Fig. 3, are parameterized as a function of ϕ using the equation below [4, 8]:

$$\chi = \frac{\psi}{\left(1-v\phi^2\right)}-\beta\left(1+2\phi\right) \tag{S6}$$

The fitted values of ψ, v, and β are listed in Supplementary Table 1.

For the ternary case, it is assumed that small fractions of the α-pinene- and limonene-derived SOMs are hydrophobic ($a_1 = \phi_1 / (\phi_1 + \phi_2)$ = 5-7%; Supplementary Table 1). This assumption is based on the experimental results that ~93% of the α-pinene-derived SOM is water soluble, and ~7% is water insoluble but methanol soluble, following the mass spectrometry analysis described in Kuwata and Lee [9]. We further assume that the interaction parameter of hydrophobic organic component with water has a large value χ_{01}, while the interaction parameter between hydrophobic and hydrophilic components χ_{12} is relatively small (Supplementary Table

1). When the hydrophobic and hydrophilic components are treated as a single compound ($\phi = \phi_1 + \phi_2$), the equivalent interaction parameter of total SOM with water can be derived by comparing Eq. S5 with Eq. S4:

$$\chi = a_1\chi_{01} + \left(1 - a_1\right)\chi_{02} - a_1\left(1 - a_1\right)\chi_{12} \quad \text{(S7)}$$

The interaction parameter χ_{02} between hydrophilic component and water can be calculated from the values of χ, χ_{01}, χ_{12} and a_1 (Supplementary Table 1).

1.2 Liquid-liquid Phase Separation

For a binary mixture, miscibility gap and co-existence of two liquid phases with different mixing ratios can occur when there is a strong interaction between solute and solvent[4]. Such behavior, however, is not predicted based on the χ values derived from experiments. For a multicomponent system, liquid-liquid phase separation (LLPS) states more generally occur when a separated two-phase configuration has a free energy lower than a miscible single-phase configuration. This general case of LLPS can be more applicable to the multicomponent laboratory SOM and ambient aerosol particles. In the present study, a minimal representation of the LLPS state requires a ternary system.

The LLPS algorithm, implemented in the present study in MATLAB, are similar to that presented in ref[10]. For a series of water-to-organic ratios, values of the free energy density function (Eq. S1) were calculated assuming a two-phase configuration. For each compounds, partition between two liquid phases was calculated by minimizing the total free energy using a Differential Evolution algorithm[10]. The initial guess was taken from the optimized results of the previous step, which had a similar water-to-organic ratio. To further minimize errors associated with initial guesses, calculations were performed for both increasing and decreasing water-to-organic ratios, and the results with lower free energy were taken. The algorithm found identical

composition for the two phases when the single-phase configuration was stable. LLPS states can be identified by the significant different compositions between two phases resulted from the optimization. As a validation, water activities were calculated for both phases using Eq. S3, and the values were always identical for a fixed water-to-organic ratio.

1.3 Surface Tension and the Köhler Theory

The treatment of surface tension generally follows the method described in ref.[6]. Surface tension σ represents an additional Gibbs free energy for a solution. The σ values of pure organic liquids are typically in the range of 0.020 – 0.065 N m^{-1} [11], lower than the σ value of pure water (0.072 N m^{-1}). At room temperature, the typical σ values for organic acids range from 0.025 to 0.040 N m^{-1} [12, 13, 14]. In the present work, a σ_{org} value of 0.035 N m^{-1} was used for both hydrophobic and hydrophilic organic components for simplicity. The effective surface tension of a liquid phase was calculated as the volume-weighted mean of σ values of the components. In the case of no LLPS (model I), this treatment is equivalent to an assumption of no bulk-surface partitioning, i.e., the surface concentration of organic solute is the same as the bulk concentration, and the effective surface tension is usually close to that of pure water at the point the CCN activation. In the presence of LLPS (model II), however, surface-active, hydrophobic organic compounds are concentrated at the droplet surface, which substantially reduces the surface tension. Sensitivity analysis shows that changing the σ_{org} value from 0.025 to 0.040 N m^{-1} can change the modeled κ_{CCN} value maximum by up to 15% in Model II, which is comparable to the uncertainty of the CCN measurements (Supplementary Fig. 5). Changing the σ_{org} value does not alter the modeled κ_{CCN} in Model I, because surface concentration of organic solute gets strongly diluted at the condition of CCN activation.

In the presence of LLPS, the organic-rich phase having a lower surface tension tends to occupy the shell of the particle to minimize the total free energy. For a growing droplet, however, the organic-rich phase may eventually not fully cover the droplet surface, because a minimum thickness δ, corresponding to a molecular single layer, is required. In the present study, a δ value of 0.3 nm was used (Supplementary Table 1). As an important adjustable parameter, the δ value used herein was comparable to the values used in recent literature[5, 6]. For the partial coverage case, the effective surface tension σ_{eff} was computed as a surface-area-weighted mean of both phases.

Combining water activity a_{w} derived from the Flory-Huggins model with the effective surface tension σ_{eff}, the Köhler curve can be expressed as:

$$S = a_w \exp\left(\frac{4\sigma_{eff} M_w}{\rho_w RTD} \right) \quad \text{(S8)}$$

where M_{w} and ρ_{w} represent the molecular weight and material density of water, respectively. D is the wet diameter of the droplet, and S is the saturation ratio. Supersaturation can be calculated as $ss = (S\text{-}1)\times 100\%$. The critical supersaturation s_c is defined as the maxima of the Köhler curve.

Supplementary References

1. Flory PJ. Thermodynamics of High Polymer Solutions. *J Chem Phys* **10**, 51-61 (1942).

2. Huggins ML. Solutions of Long Chain Compounds. *J Chem Phys* **9**, 440-440 (1941).

3. Dormidontova EE. Role of Competitive PEO−Water and Water−Water Hydrogen Bonding in Aqueous Solution PEO Behavior. *Macromol* **35**, 987-1001 (2002).

4. Petters MD, *et al.* Cloud droplet activation of polymerized organic aerosol. *Tellus B* **58**, 196-205 (2006).

5. Ruehl CR, Davies JF, Wilson KR. An interfacial mechanism for cloud droplet formation on organic aerosols. *Science* **351**, 1447-1450 (2016).

6. Ovadnevaite J, *et al.* Surface tension prevails over solute effect in organic-influenced cloud droplet activation. *Nature* **546**, 637 (2017).

7. van der Sman RGM. Predicting the solubility of mixtures of sugars and their replacers using the Flory-Huggins theory. *Food & Function* **8**, 360-371 (2017).

8. Wolf BA. Chain Connectivity and Conformational Variability of Polymers: Clues to an Adequate Thermodynamic Description of Their Solutions, 2. *Macromol Chem Phys* **204**, 1381-1390 (2003).

9. Kuwata M, Lee W-C. 1-octanol-water partitioning as a classifier of water soluble organic matters: Implication for solubility distribution. *Aerosol Sci Tech* **51**, 602-613 (2017).

10. Zuend A, Marcolli C, Peter T, Seinfeld JH. Computation of liquid-liquid equilibria and phase stabilities: implications for RH-dependent gas/particle partitioning of organic-inorganic aerosols. *Atmos Chem Phys* **10**, 7795-7820 (2010).

11. Bormashenko E. Why are the values of the surface tension of most organic liquids similar? *Am J Phys* **78**, 1309-1311 (2010).

12. Álvarez E, Vázquez G, Sánchez-Vilas M, Sanjurjo B, Navaza JM. Surface Tension of Organic Acids + Water Binary Mixtures from 20 °C to 50 °C. *Journal of Chemical & Engineering Data* **42**, 957-960 (1997).

13. Chumpitaz LDA, Coutinho LF, Meirelles AJA. Surface tension of fatty acids and triglycerides. *Journal of the American Oil Chemists' Society* **76**, 379-382 (1999).

14. Riipinen I, *et al.* Adipic and Malonic Acid Aqueous Solutions: Surface Tensions and Saturation Vapor Pressures. *J Chem Phys A* **111**, 12995-13002 (2007).

15. Renbaum-Wolff L, *et al.* Observations and implications of liquid–liquid phase separation at high relative humidities in secondary organic material produced by α-pinene ozonolysis without inorganic salts. *Atmos Chem Phys* **16**, 7969-7979 (2016).

16. Song M, Liu P, Martin ST, Bertram AK. Liquid–liquid phase separation in particles containing secondary organic material free of inorganic salts. *Atmos Chem Phys* **17**, 11261-11271 (2017).

17. Chen Q, Liu Y, Donahue NM, Shilling JE, Martin ST. Particle-phase chemistry of secondary organic material: modeled compared to measured O:C and H:C elemental ratios provide constraints. *Environmental Science & Technology* **45**, 4763-4770 (2011).

18. Kuwata M, Zorn SR, Martin ST. Using elemental ratios to predict the density of organic material composed of carbon, hydrogen, and oxygen. *Environ Sci Technol* **46**, 787-794 (2012).

19. Norrish RS. An equation for the activity coefficients and equilibrium relative humidities of water in confectionery syrups. *Int J Food Sci Technol* **1**, 25-39 (1966).

20. Zobrist B, *et al.* Ultra-slow water diffusion in aqueous sucrose glasses. *Phys Chem Chem Phys* **13**, 3514-3526 (2011).

21. Starzak M, Peacock SD. Water activity coefficient in aqueous solutions of sucrose-a comprehensive data analysis. *Zuckerindustrie* **122**, 380-388 (1997).

	Model I		Model II[a]				Water activity of LLPS	
SOM type	N	Fitted parameters for $\chi(\phi)$	Initial hydrophobic fraction a_1	χ_{12}	χ_{01}	δ (nm)	Predicted	Measured
α-pinene + O_3	14	ψ = 0.02105<br>v = 0.7632<br>β = -0.3400	0.07	0.1	1.5	0.3	0.9498 – 0.9999	> 0.950[b]
limonene + O_3	14	ψ = 0.3697<br>v = 0.4533<br>β = -0.0486	0.05	0.1	1.45	0.3	0.9550 – 0.9998	> 0.987[c]

Supplementary Table 1. Summary of Flory-Huggins-based thermodynamic models. [a]Ternary model assumes both hydrophobic and hydrophilic organic components have the same molecular volume. The molecular volume ratio N values are the same as that used in the binary model. The values of interaction parameter χ_{02} (not listed) were calculated from the values of χ, χ_{12}, χ_{01} and a_1 using Eq. S7, such that the overall interaction parameter for hydrophobic and hydrophilic components with water is consistent with $\chi(\phi)$ values fitted from experiments. [b]Value taken from ref.[15]. [c]Value taken from ref.[16]

Precursor	Oxidation pathway	Precursor concentration (ppb)	Mass concentration (μg m^{-3})[a]	O:C[b]	H:C[b]	Material density (kg m^{-3})[c]
α-pinene	O_3	200	60 - 110	0.43	1.70	1.19×10^3
limonene	O_3	70	60 - 90	0.59	1.54	1.34×10^3
toluene	OH	200	70 - 100	1.08	1.69	1.55×10^3
dodecane	OH	50	60 - 80	0.47	1.69	1.22×10^3

Supplementary Table 2. Precursor, oxidant, organic particle mass concentration, elemental ratios, and material densities for the different types of SOM studied herein. [a]Values derived from number-diameter distributions measured by a Scanning Mobility Particle Sizer (SMPS). Mass concentrations were calculated from volume concentration using the listed material density. [b]Values derived from analysis of mass spectra recorded by a High-Resolution Time-of-Flight Aerosol Mass Spectrometer (HR-TOF-AMS). Analysis was based on the explicit approach described by Chen et al.[17]. [c]Values calculated from the O:C and H:C ratios using the method of Kuwata et al.[18].

List of Supplementary Figures

Supplementary Figure 1. (a) Conceptual framework of hygroscopic growth of a secondary organic aerosol particle based on the assumption of solubility limits. The dry particle is composed of organic compounds with different degrees of solubility. The wet particle consists of an insoluble organic core and an aqueous shell with organics dissolved in water. (b) Conceptual framework of hygroscopic growth of an amorphous organic particle undertaken gradual deliquescence. Organics and water form one uniform amorphous phase at low RH. The concentration of a single organic compound may exceed its solubility limit. Liquid-liquid phase separation can occur at high RH for some types of secondary organic material.

Supplementary Figure 2. Mass of water m_{water} taken up by the thin film as a function of film dry mass m_{dry}. m_{water} shown at m_{dry} = 0 represents water adsorption on the clean SiO_2 surface of the QCM sensor.

Supplementary Figure 3. Flory-Huggins interaction parameter χ derived from fitting of hygroscopic growth data. (a) α-pinene-derived SOM and (b) limonene-derived SOM.

Supplementary Figure 4. Mass-based hygroscopic growth factor g_m measured for amorphous sucrose thin films for method validation. Data from literature are shown for comparison[19, 20, 21]. Dashed line illustrates the idealized deliquescence behavior for crystalline sucrose.

Supplementary Figure 5. Sensitivity analysis of κ_{CCN} on different values of pure organic surface tension σ_{org}. The κ_{CCN} values are calculated for α-pinene-derived SOM using the LLPS model (Model II, see main text) by assuming different σ_{org} values within a typical range. κ_{CCN} values derived from the size-resolved CCN measurements are shown for comparison.

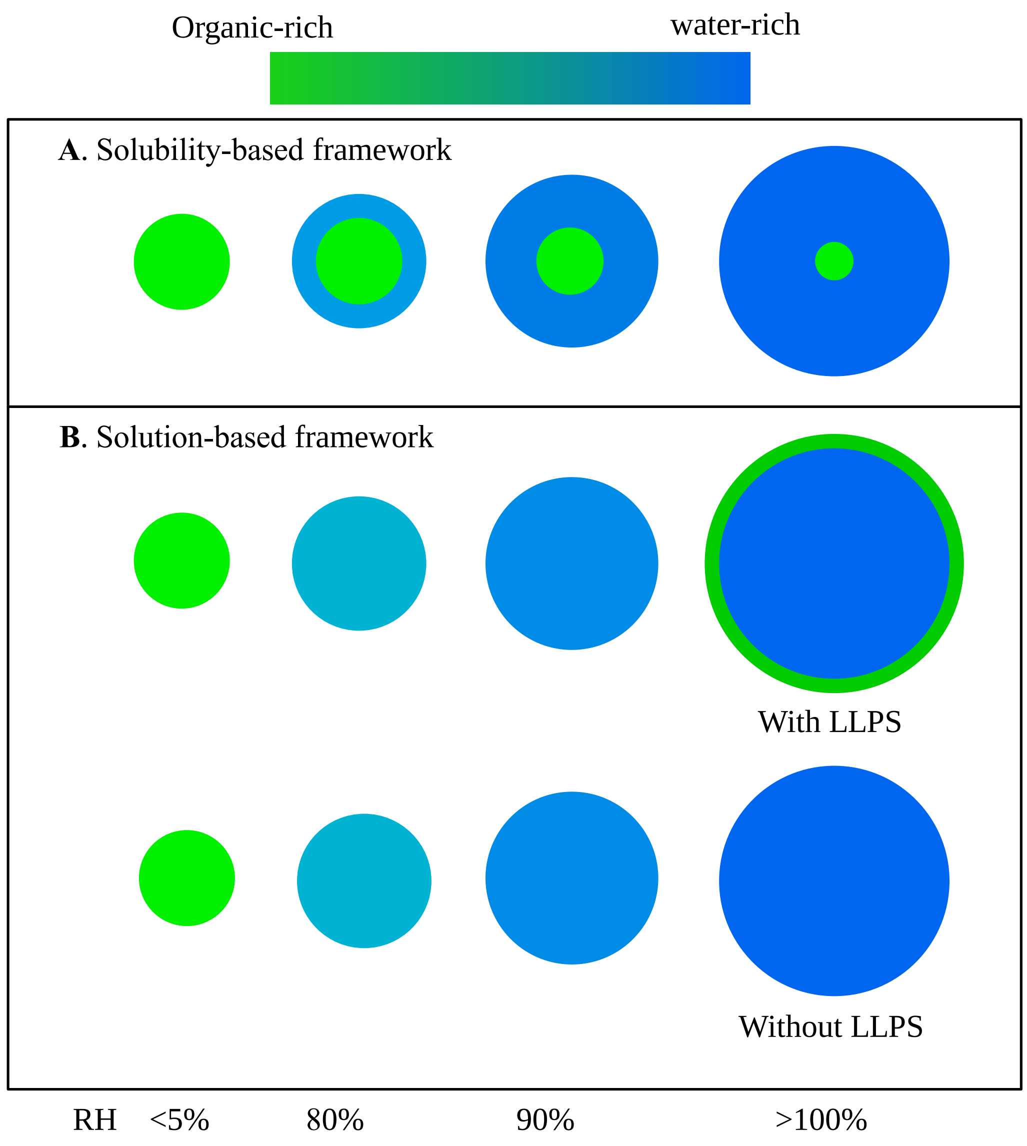

Supplementary Figure 1 (A) Conceptual framework of hygroscopic growth of a secondary organic aerosol particle based on the assumption of solubility limits. The dry particle is composed of organic compounds with different degrees of solubility. The wet particle consists of an insoluble organic core and an aqueous shell with organics dissolved in water. **(B) Conceptual framework of hygroscopic growth of an amorphous organic particle undertaken gradual deliquescence.** Organics and water form one uniform amorphous phase at low RH. The concentration of a single organic compound may exceed its solubility limit. Liquid-liquid phase separation can occur at high RH for some types of secondary organic material.

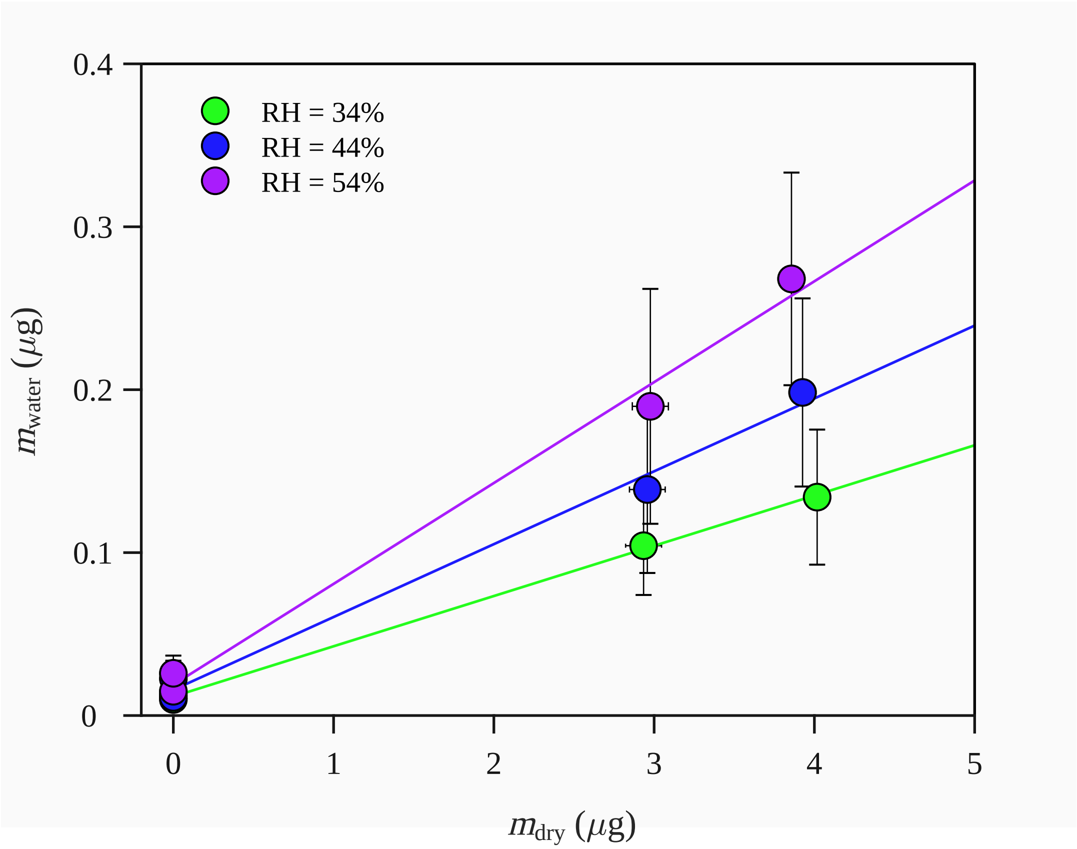

Supplementary Figure 2 Mass of water m_{water} taken up by the thin film as a function of film dry mass m_{dry}. m_{water} shown at $m_{dry} = 0$ represents water adsorption on the clean SiO_2 surface of the QCM sensor.

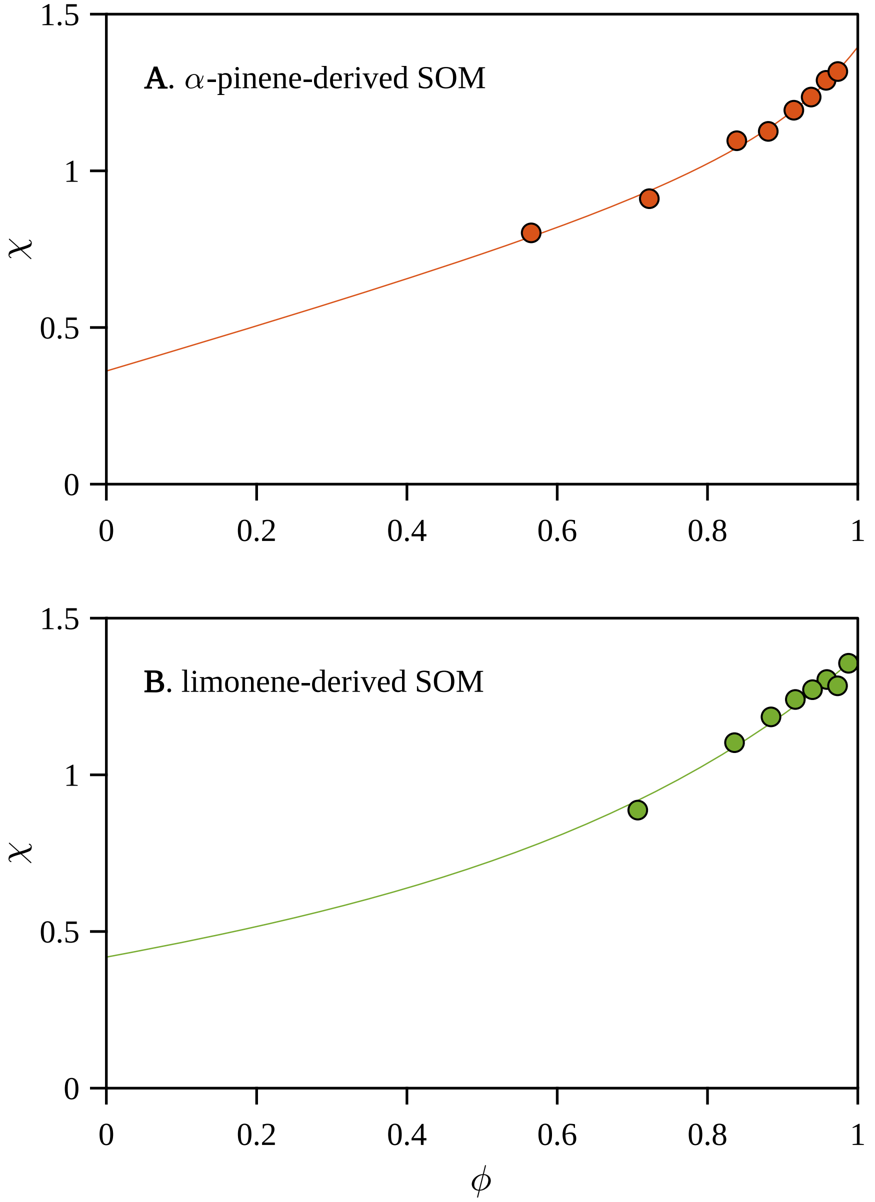

Supplementary Figure 3 Flory-Huggins interaction parameter χ derived from fitting of hygroscopic growth data. (a) α-pinene-derived SOM and (b) limonene-derived SOM.

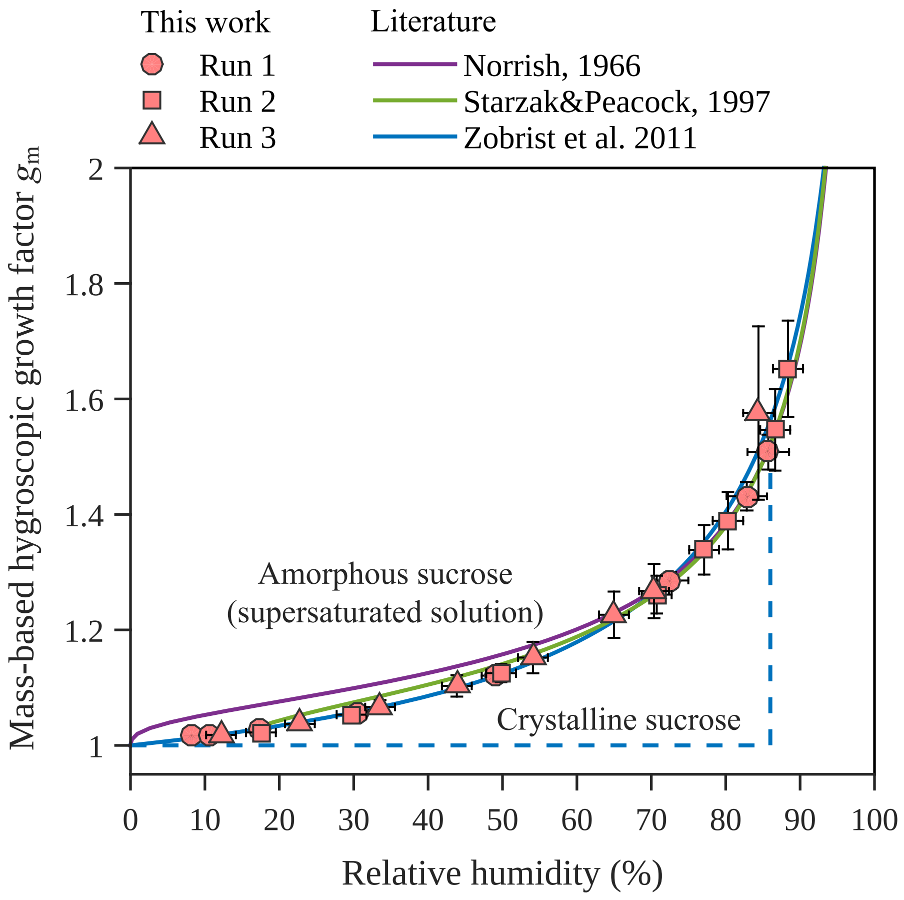

Supplementary Figure 4 Mass-based hygroscopic growth factor gm measured for amorphous sucrose thin films for method validation. Data from literature are shown for comparison[19, 20, 21]. Dashed line illustrates the idealized deliquescence behavior for crystalline sucrose.

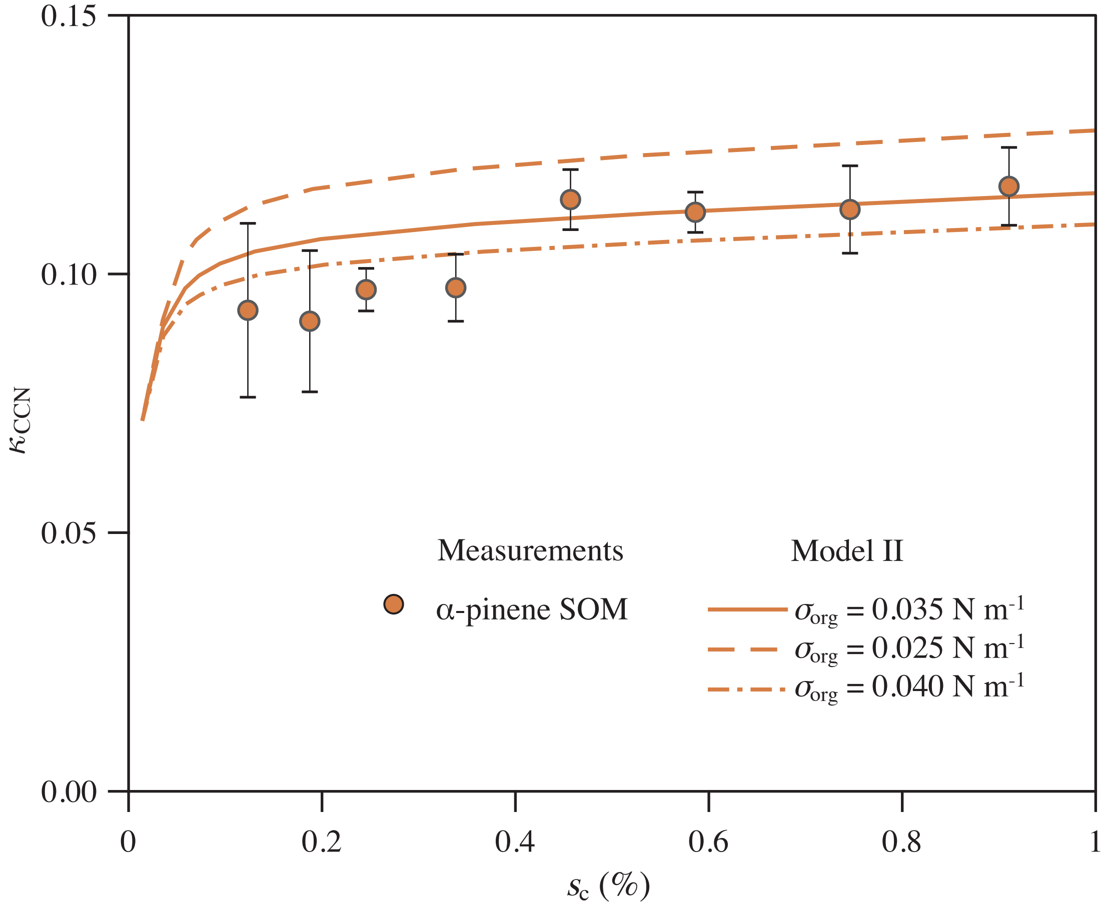

Supplementary Figure 5 Sensitivity analysis of κ_{CCN} on different values of pure organic surface tension σ_{org}. The κ_{CCN} values are calculated for α-pinene-derived SOM using the LLPS model (Model II, see main text) by assuming different σ_{org} values within a typical range. κCCN values derived from the size-resolved CCN measurements are shown for comparison.
